# Association of LH rising before luteal support and reproductive outcomes in artificial frozen embryo transfer cycles

**DOI:** 10.3389/fendo.2026.1886113

**Published:** 2026-07-23

**Authors:** Ming-Hsuean Chien, Tsung-Hsuan Lai

**Affiliations:** 1 Division of Reproductive Endocrinology and Infertility, Department of Obstetrics and Gynecology, Cathay General Hospital, Taipei, Taiwan; 2 Department of Obstetrics and Gynecology, Cathay General Hospital, Hsinchu Branch, Hsinchu, Taiwan; 3 School of Medicine, Fu-Jen Catholic University, New Taipei City, Taiwan

**Keywords:** artificial cycles, endometrial receptivity, frozen embryo transfer, LH rising, reproductive outcomes

## Abstract

**Introduction:**

In natural cycles, rising estradiol (E2) suppresses pituitary luteinizing hormone (LH) secretion until a threshold is reached, triggering the LH surge and ovulation. During artificial-cycle frozen embryo transfer (AC-FET), prolonged E2 exposure may induce endogenous LH elevation before progesterone administration. Limited studies have examined the impact of LH dynamics on reproductive outcomes in AC-FET cycles. This study investigated the association between LH rising before luteal support and reproductive outcomes and further explored the relative contributions of basal LH, and pre-P LH.

**Methods:**

This retrospective cohort study included patients aged 25–44 years undergoing D3 or D5 AC-FET without GnRH agonist suppression. A total of 582 cycles were categorized according to the LH rising ratio, defined as pre-progesterone LH divided by basal LH. Cycles were classified as LH rising ratio <3 (group 1) or ≥3 (group 2). Outcomes included implantation (IR), clinical pregnancy (CPR), ongoing pregnancy (OPR), and abortion rates (AR).

**Results:**

In D3 embryo transfer cycles, an LH rising ratio ≥3 was associated with lower IR (21.1% vs. 13.2%; OR 0.57, 95% CI 0.38–0.82) and CPR (43.3% vs. 28.4%; OR 0.52, 95% CI 0.31–0.87). AR was higher and OPR lower, although not statistically significant. Similar trends were observed in D5 cycles without statistical significance. After adjustment using multivariable generalized estimating equation models, the associations in D3 cycles remained significant. However, decoupling analyses suggested that the LH rising ratio primarily functioned as a surrogate marker of lower basal LH rather than an independent effect of elevated pre-P LH, as basal LH remained associated with implantation outcomes whereas pre-P LH did not. Quartile analyses did not demonstrate a threshold effect for either basal LH or pre-P LH.

**Conclusion:**

A higher LH rising ratio before progesterone supplementation was associated with reduced implantation and clinical pregnancy rates in D3 AC-FET cycles. Additional analyses suggest that the LH rising ratio may primarily serve as a surrogate marker of lower basal LH rather than an independent effect of elevated pre-P LH. The relationship between LH dynamics and reproductive outcomes remains complex and may not be fully explained by basal or pre-P LH alone.

## 1 Introduction

Frozen embryo transfer (FET) was first introduced in the 1980s with the first birth reported in 1984 (1). It was not until after 2000, when vitrification techniques matured, that it became widely adopted as a safe and effective practice. FET is performed using different cycle regimens: true natural cycles (NC-FET); modified NC-FET; artificial cycles in which the endometrium is prepared by estrogen (E2) and progesterone (P) hormones (AC-FET), while AC-FET can be used with or without a gonadotrophin-releasing hormone agonist (GnRHa); and ovulation induction FET cycles in which ovulation is induced by gonadotropins (2).

A recent Cochrane review comparing various cycle regimens and endometrial preparation methods for FET had found insufficient evidence to recommend one specific approach over another (2). However, the use of exogenous E2 and P in AC-FET offers advantages in facilitating cycle control and providing flexibility in scheduling FET (3).

In AC-FET, both fixed-dose regimens (oral E2 6-12 mg/day) and step-up protocols (gradually increasing the dose from 2 to 8 mg over approximately 10–15 days to mimic natural hormonal change) are commonly used. While both approaches are effective, a moderate step-up regimen starting at around 4-6 mg may offer slightly improved endometrial development and pregnancy outcomes (4).

Interestingly, it had been observed that in AC-FET cycles without pituitary suppression, initiating E2 supplementation from the early cycle day may induce an elevation of luteinizing hormone (LH) level by positive feedback on the hypothalamus–pituitary–ovarian axis (H–P–O axis) during endometrial preparation, resembling the pre-ovulatory LH surge seen in natural cycles. However, adequate E2 level typically inhibits LH elevation from negative feedback. In AC-FET cycles, the LH rise occurs without accompanying ovulation or an increase in progesterone concentration (5, 6).

Previous studies by Griesinger et al. and Khoury et al. found that there was no significant association between LH levels on the day before progesterone supplementation (pre-P day) or dynamic LH rises and pregnancy outcomes in AC-FET cycles (7, 8). Afterward, three large cohort studies stratifying LH level by quartile showed that low LH levels (<25th percentile) before progesterone initiation was significantly associated with poorer implantation and higher quartile of LH levels have better pregnancy outcomes (9–11). However, these studies stratifying LH level by quartile in total IVF cycles did not reflect the real change of LH per cycle in each patient. Therefore, we considered that the LH rising ratio, defined as the LH concentration on the day before progesterone supplementation divided by the basal LH level for each individual, more accurately reflects the true dynamic change of LH in AC-FET cycles.

Given the limited and inconclusive evidence regarding the impact of LH ratio elevation prior to progesterone supplementation, we conducted this study to evaluate whether excessive endogenous LH rising before P supplementation in AC-FET cycles affect reproductive outcomes.

## 2 Material and methods

### 2.1 Study design and study population

A total of 1,093 AC-FET cycles at the Assisted Reproductive Center in Cathay General Hospital were enrolled in this retrospective study from January 2016 to October 2024. The study was approved by the Cathay General Hospital IRB (No. CGH-P114016).

In this study, the inclusion criteria were (a) cases involving autologous FET without GnRH agonist suppression; (b) participants had to be the cases of AC-FET; (c) participants had to be the cases involving D3 or D5 FET. The exclusion criteria were (a) participants whose age <25 years old or >44 years; (b) participants using donated oocytes; (c) participants with the presence of ovarian cysts identified on cycle day 2 or 3; (d) participants with pertinent data missing; (e) participants with unordinary laboratory data such as basal P levels >1 ng/ml or basal E2 levels >100 pg/ml; (f) any premature rise in serum P before progesterone supplementation.

**Figure 1:**
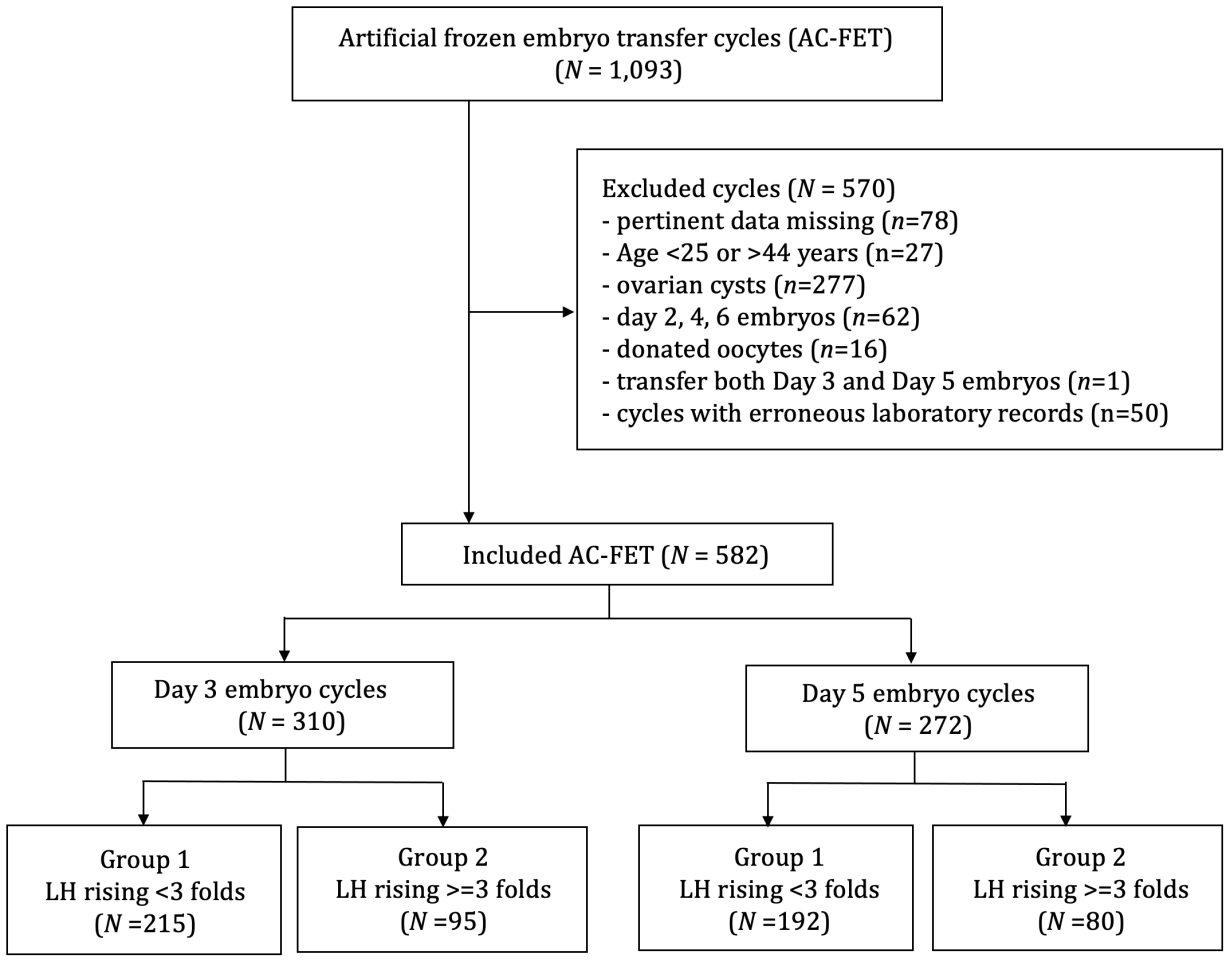
Flowchart of the study. *AC-FET, artificial cycle-frozen embryo transfer.

A total of 78 cycles were excluded because of missing data, and 27 cycles were excluded because the patients were younger than 25 years or older than 44 years. A total of 277 cycles were excluded because ovarian cystic lesions ≥15 mm were identified on cycle day 2 or 3. A total of 62 cycles involving D2, D4, or D6 embryo transfer and one cycle involving sequential transfer of both D3 and D5 embryos were excluded. There were 16 cycles using donated oocytes that were also excluded. In addition, 50 cycles containing implausible laboratory values attributable to apparent transcription or data-entry errors were excluded during data cleaning. These exclusions were not based on LH-related measurements. Ultimately, 582 AC-FET cycles were included in the final analysis, comprising 310 D3 embryo transfer cycles and 272 D5 embryo transfer cycles (**Figure 1**).

### 2.2 Grouping

A total of 582 candidates were categorized into two groups based on the LH rising ratio as the LH level on the pre-P day divided to the basal LH level in each patient. A ratio of LH ≥ 3 was defined as an excessive LH elevation in this study. Because no universally accepted cutoff has been established for the LH rising ratio in AC-FET cycles, a threefold increase was selected *a priori* as a pragmatic categorization of substantial LH elevation. LH rising ratio <3 is assigned as group 1, and LH ratio ≥3 is assigned as group 2. The case numbers of each group according to the day of embryo transfer were as the following: D3 group 1 (n= 215), D3 group 2 (n= 95), D5 group 1 (n= 192), and D5 group 2 (n= 80) (**Figure 1**).

### 2.3 AC-FET protocols

Patients were administered estradiol valerate (Estrade 2 mg, Synmosa, Hsinchu, Taiwan) at a dose of 8 mg daily from cycle day 3 for 7 days and then 12 mg daily for 7 days for endometrial priming, followed by a maintenance dose of 8 mg daily after progesterone supplementation. Oral P (Utrogestan 100 mg, Olic, Ayutthaya, Thailand) two capsules thrice daily and transvaginal P (Crinone^®^ 8%; Merck Serono, Hertfordshire, UK) one applicator twice daily were given for luteal phase support for 14 days. Serum β-human chorionic gonadotropin was measured 14 days after FET. If β-hCG was positive, E2 and progesterone supplementation was continued until 12 weeks of gestation.

### 2.4 Study outcomes and definitions

The primary endpoints are implantation rate (IR), clinical pregnancy rate (CPR), ongoing pregnancy rate (OPR), and abortion rate (AR). IR is defined as the percentage of cycles that have visualization of a gestational sac in uterine cavity via ultrasound divided by the number of embryos transferred. CPR is defined as pregnancies with the presence of fetal heart beats via ultrasound divided by the number of embryo transfer cycles. OPR is defined as the percentage of successful pregnancy surviving after 20 weeks of gestation divided by the number of embryo transfer cycles. AR is defined as the percentage of pregnancies with spontaneous loss before 20 completed weeks of gestational age divided by the number of implantations. In addition, serum E2, P, and LH levels were measured on cycle day 2 (basal hormones) and the day before progesterone supplementation (pre-P hormones). Hormone measurements were obtained during routine clinical visits at the morning according to standard clinical practice. Although hormone measurements were generally obtained during routine morning visits, exact blood sampling times were not available in the retrospective database. Reproductive outcomes were assessed including IR, CPR, OPR, and AR.

### 2.5 Statistical analysis

Statistical analyses were performed using IBM SPSS Statistics for Windows, Version 27.0 (IBM Corp., Armonk, NY, USA). Continuous variables were presented as mean ± standard deviation (SD) and compared using the Student’s t-test or one-way analysis of variance (ANOVA), as appropriate. Categorical variables were compared using the chi-square test or Fisher’s exact test.

Generalized estimating equation (GEE) models with an exchangeable working correlation structure and robust standard errors were used to account for within-patient correlation. Implantation rate was analyzed using a binomial GEE model with the number of gestational sacs specified as events and the number of embryos transferred specified as trials. Clinical pregnancy rate, ongoing pregnancy rate, and abortion rate were analyzed using binomial GEE models with a logit link function. A multivariable GEE analysis was performed to assess the association between LH rising ratio and reproductive outcomes. Separate multivariable GEE models were constructed for D3 and D5 cycles. Covariates for each model were selected based on baseline variables that differed significantly between the comparison groups and were considered clinically relevant. The models were adjusted for confounders including age, BMI, basal LH, basal P, basal FSH, Pre-P E2, Pre-P P, endometrial thickness, the number of embryos transferred for D3 cycles, and age, BMI, AMH, basal E2, basal LH, basal P, Pre-P E2, Pre-P P, endometrial thickness, and the number of embryos transferred for D5 cycles.

Crude odds ratios (ORs) and adjusted odds ratios (aORs) with 95% confidence intervals (CIs) were calculated. Adjusted ORs were estimated using the stage-specific multivariable GEE models described above for D3 and D5 cycles, respectively. To assess whether the association between LH rise and reproductive outcomes differed according to embryo stage, an interaction term between embryo stage and LH-rise group was included in the pooled GEE model.

Sensitivity analyses were performed using alternative LH-rise thresholds of twofold and fourfold. In addition, the LH rising ratio was analyzed as a continuous variable in GEE models to evaluate the association between LH dynamics and reproductive outcomes without reliance on predefined cutoffs. Basal LH and pre-P LH were also analyzed as separate continuous covariates to explore their independent associations with reproductive outcomes. Additional exploratory analyses were performed using quartiles of basal LH and pre-P LH concentrations. Multicollinearity among predictors was assessed using variance inflation factors (VIFs).

No adjustment for multiple comparisons was performed because the evaluated outcomes were predefined, clinically related reproductive endpoints and the analyses were hypothesis-driven rather than exploratory in nature.

## 3 Results

### 3.1 Descriptive analysis

The baseline factors including age, BMI, AMH, basal hormones (E2, LH, P, FSH), Pre-P hormones (E2, LH, P), endometrial thickness, ET day hormones (E2, P), the numbers of embryo transferred, and reproductive outcomes were calculated. Group 2 in D3 FET cycles had a lower BMI, basal LH, and basal FSH level (P<0.05), whereas there were higher levels of basal P and pre-P LH (P<0.05). There were no significant differences in age, AMH, endometrial thickness, and ET number between the groups. In terms of D5 FET cycles, lower BMI, AMH, basal LH, and pre-P E2 levels were found in the group 2; whereas higher basal E2, basal P and pre-P LH levels were found in the group 2 (P<0.05). There were no significant differences in age, endometrial thickness, and ET number (**Table 1**).

**Table 1:** Baseline demographics and cycle characteristics of the study.

Baseline demographics	D3 embryo cycles (N = 310)			D5 embryo cycles (N = 272)		
	Group 1 (N = 215)	Group 2 (N = 95)	P value	Group 1 (N = 192)	Group 2 (N = 80)	P value
Maternal age (years)	38.68 ± 3.85	38.22 ± 3.34	0.290	35.46 ± 4.06	35.44 ± 3.94	0.961
Maternal BMI (kg/m^2^)	23.32 ± 3.89	22.18 ± 3.48	0.012^*^	23.68 ± 4.19	22.30 ± 3.84	0.010^*^
AMH (ng/mL)	3.21 ± 3.44	3.06 ± 2.31	0.650	6.19 ± 5.03	4.46 ± 3.47	0.001^*^
Basal E2 (pg/mL)	32.27 ± 18.73	33.90 ± 18.05	0.471	30.42 ± 15.02	36.84 ± 15.92	0.003^*^
Basal LH (mIU/mL)	5.49 ± 2.55	3.84 ± 2.01	0.000^*^	6.07 ± 2.89	4.61 ± 2.37	0.000^*^
Basal P4 (ng/mL)	0.23 ± 0.15	0.30 ± 0.21	0.004^*^	0.24 ± 0.15	0.31 ± 0.20	0.002^*^
Basal FSH (mIU/mL)	8.58 ± 4.07	7.14 ± 2.45	0.000^*^	7.06 ± 2.73	6.81 ± 2.59	0.472
Pre-P E2 (pg/mL)	397.91 ± 184.80	341.90 ± 145.85	0.005	389.54 ± 159.31	343.21 ± 153.47	0.026^*^
Pre-P LH (mIU/mL)	8.75 ± 5.74	17.82 ± 11.12	0.000^*^	9.34 ± 5.13	20.82 ± 10.26	0.000^*^
Pre-P P4 (ng/mL)	0.14 ± 0.13	0.14 ± 0.13	0.851	0.14 ± 0.12	0.15 ± 0.11	0.291
LH rising multiple	1.60 ± 0.74	5.34 ± 3.45	0.000^*^	1.61 ± 0.68	5.95 ± 5.77	0.000^*^
Endometrial thickness (mm)	10.64 ± 1.66	10.66 ± 1.44	0.926	10.60 ± 1.48	10.88 ± 1.59	0.172
ET* day E2 (pg/mL)	189.24 ± 109.32	204.29 ± 136.14	0.344	180.83 ± 104.41	186.73 ± 98.47	0.662
ET* day P4 (ng/mL)	47.80 ± 19.12	44.39 ± 20.36	0.169	46.04 ± 19.14	49.83 ± 32.37	0.334
Number of embryos been transferred	2.27 ± 0.82	2.46 ± 0.87	0.069	1.61 ± 0.73	1.64 ± 0.66	0.801

*Statistical method: independent t-test.

*Values are presented as mean ± standard deviation (SD) for normally distributed variables and median (interquartile range [IQR]) for non-normally distributed variables. *P* < 0.05 was considered statistically significant.

BMI, body mass index; AMH, anti-Mullerian hormone; E2, estradiol; LH, luteinizing hormone; P4, progesterone; FSH, follicle-stimulating hormone; pre-P, the day before progesterone supplementation; ET, embryo transfer.

### 3.2 Pregnancy outcomes

The final cohort comprised 582 AC-FET cycles contributed by 393 women. Most women contributed a single cycle (n=255), whereas 102 women contributed two cycles, 25 contributed three cycles, and 11 contributed four or more cycles. The D3 subgroup included 310 cycles from 236 women, whereas the D5 subgroup included 272 cycles from 212 women.

In the D3 embryo cycles, IR and CPR were lower in cycles with ≥3-fold LH elevation (21.1% vs 13.2%, P = 0.003 < 0.05, 0.57 [0.38-0.82]), (43.3% vs 28.4%, P = 0.013 <0.05, 0.52 [0.31-0.87]). AR was higher and OPR was lower in cycles with ≥3-fold LH elevation, but with no significance (4.9% vs 6.5%, P = 0.656, 1.35 [0.30-6.67], 33.0% vs 24.2%, P = 0.108, 0.64 [0.38-1.10]). In the D5 embryo cycles, IR, CPR, and OPR were lower in cycles with ≥3-fold LH elevation, but with no significance. AR was higher in cycles with ≥3-fold LH elevation, but with no significance (**Table 2**).

**Table 2:** Clinical reproductive outcomes of the study.

Reproductive outcomes, no. (%)	D3 embryo cycles (N = 310)				D5 embryo cycles (N = 272)			
	Group 1 (N = 215)	Group 2 (N = 95)	OR (95% CI)	P value	Group 1 (N = 192)	Group 2 (N = 80)	OR (95% CI)	P value
Implantation	103/488 (21.1%)	31/234 (13.2%)	0.57 (0.38-0.82)	0.003^*^	86/310 (27.7%)	30/131 (22.9%)	0.78 (0.48-1.30)	0.355
Clinical pregnancy	93/215 (43.3%)	27/95 (28.4%)	0.52 (0.31-0.87)	0.013^*^	81/192 (42.2%)	27/80 (33.8%)	0.71 (0.40-1.24)	0.225
Abortion rate	5/103 (4.9%)	2/31 (6.5%)	1.35 (0.30-6.67)	0.656	3/86 (3.5%)	3/30 (10%)	3.06 (0.55-14.29)	0.212
Ongoing pregnancy rate	71/215 (33.0%)	23/95 (24.2%)	0.64 (0.38-1.10)	0.108	58/192 (30.2%)	22/80 (27.5%)	0.87 (0.48-1.59)	0.654

*Statistical method: Generalized estimating equations (GEE).

*The denominators of clinical pregnancy rate and ongoing pregnancy rate were the embryo transfer cycle numbers, and the denominator of the implantation rate was the total numbers of embryos transferred. The denominator of abortion rate was the number of patients that have the presence of a gestational sac under ultrasound.

^*^ p < 0.05 considered statistically significant.

A multivariate GEE analysis was calculated to assess the effect of LH rising multiple and reproductive outcomes; after adjusting for confounders, ≥3-fold LH elevation is still significantly associated with lower IR and CPR, respectively, but with less significance, and only in D3 cycles (adjusted odds ratio: 0.60 [0.41–0.87], P = 0.008 <0.05), (adjusted odds ratio: 0.56 [0.34–0.95], P = 0.031 < 0.05) (**Table 3**).

**Table 3:** Adjusted odds ratio of clinical reproductive outcomes of the study.

Reproductive outcomes	D3 embryo cycles (N = 310)		D5 embryo cycles (N = 272)	
	aOR (95% CI)	P value	aOR (95% CI)	P value
Implantation rate	0.60 (0.41-0.87)	0.008^*^	0.77 (0.46-1.27)	0.302
Clinical pregnancy rate	0.56 (0.34-0.95)	0.031^*^	0.71 (0.40-1.26)	0.237
Abortion rate	1.30 (0.27-6.25)	0.741	2.86 (0.55-14.29)	0.211
Ongoing pregnancy rate	0.70 (0.41-1.21)	0.206	0.88 (0.48-1.63)	0.686

* Statistical method: multivariate generalized estimating equations (GEE).

* The denominators of clinical pregnancy rate and ongoing pregnancy rate were the embryo transfer cycle numbers, and the denominator of the implantation rate was the total numbers of embryos transferred. The denominator of abortion rate was the number of patients that have the presence of a gestational sac under ultrasound.

^*^ p < 0.05 considered statistically significant.

In a pooled GEE model including embryo stage, LH-rise group, and the embryo stage × LH-rise interaction term, no significant interaction was observed (P for interaction = 0.418).

Sensitivity analyses using alternative LH-rise thresholds of 2-fold and 4-fold yielded results generally consistent with the primary analysis, with elevated LH rise remaining associated with lower implantation and clinical pregnancy rates (**Supplementary Table 1**).

Additional decoupling analyses entering basal LH and pre-P LH as separate continuous covariates demonstrated that basal LH remained independently associated with implantation outcomes, whereas pre-P LH did not. Neither variable was independently associated with clinical pregnancy outcomes (**Supplementary Table 2**).

Additional quartile analyses of absolute LH concentrations did not demonstrate a robust and consistent threshold effect for either basal LH or pre-P LH. Although higher basal LH quartiles showed progressively increased odds of implantation, these associations did not reach statistical significance. Similarly, pre-P LH quartiles did not demonstrate consistent associations across reproductive outcomes (**Supplementary Table 3**).

Continuous-variable analyses of the LH rising ratio were also performed and are presented in **Supplementary Table 4**.

## 4 Discussion

Previous studies stratifying LH levels into quartile have shown that CPR and LBR significantly increased from the first to the fourth LH quartile (9–11). A recent study also stratifying outcomes by LH quartiles concluded that low LH levels were significantly associated with a decreased LBR and an increased risk of miscarriage (11). However, these studies stratifying LH level by quartile in total IVF cycles did not reflect the real change of LH per cycle in each patient. In our study, we defined an elevated LH rise as a pre-P LH level that was at least threefold higher than the basal LH level within the same cycle. Although no universally accepted cutoff has been established for the LH rising ratio in AC-FET cycles, a threefold increase was selected *a priori* as a pragmatic categorization of substantial LH elevation. The prespecified threefold cutoff was informed by previous studies of LH surge onset in natural menstrual cycles, in which thresholds ranging from 1.8- to 6-fold above baseline LH have been used to define surge onset (12). Because comparable thresholds have not been established in AC-FET cycles, this cutoff was selected by analogy and should be regarded as an exploratory rather than a validated threshold.

Sensitivity analyses using alternative LH rise thresholds (2-fold and 4-fold) yielded consistent results, supporting the robustness of the association between elevated LH rise and impaired reproductive outcomes. Importantly, the association remained directionally consistent across alternative LH-rise thresholds (2-fold, 3-fold, and 4-fold), indicating that the findings were not dependent on the specific cutoff selected (**Supplementary Table 1**).

This approach may provide complementary information regarding cycle-specific LH dynamics beyond analyses based solely on absolute LH concentrations. Interestingly, our results revealed that an LH rising ratio ≥3-fold before progesterone initiation was significantly associated with lower IR (adjusted 95% CI: 0.60 [0.41-0.87], P = 0.008 < 0.05) and CPR (adjusted 95% CI: 0.56 [0.34-0.95], P = 0.031 < 0.05) in AC-FET cycles. Although these associations appeared more evident in day 3 embryo transfer cycles, formal interaction testing did not support a statistically significant difference between day 3 and day 5 transfers. Our findings suggest that LH dynamics before progesterone initiation are associated with reproductive outcomes in AC-FET cycles. However, whether these associations primarily reflect dynamic LH changes, baseline LH status, or a combination of both remains uncertain.

In addition to incomplete suppression of FSH by exogenous estrogen, the premature endogenous LH rise observed in artificial FET cycles without pituitary suppression may be partly explained by low basal LH levels, whereby even a small absolute increase results in a disproportionately elevated LH ratio (8). Furthermore, intrinsic dysregulation of LH pulsatility—particularly in patients with PCOS—may contribute through increased pulse frequency and amplitude (13). The overall estradiol milieu, whether derived endogenously or through exogenous supplementation, may also modulate LH dynamics, with higher estradiol exposure potentially facilitating LH elevation prior to progesterone initiation (14).

Methodological factors should also be considered when interpreting premature LH rise in AC-FET. Given the pulsatile nature of LH secretion (15), variations in sampling timing (16), assay characteristics, and the definition of baseline LH can substantially influence the calculated LH ratio. LH exhibits pulsatile secretion and potential diurnal variation. Although hormone measurements were generally obtained during routine morning clinical visits, exact blood sampling times were not available in the retrospective database. Collectively, these findings suggest that an elevated LH ratio in artificial cycles does not necessarily suggest a pathological process but rather reflects a complex interplay among residual ovarian activity, the endocrine environment, and measurement-related factors.

The LH/choriogonadotropin receptor (LHCGR) is a G-protein–coupled receptor activated by both LH and human chorionic gonadotropin (hCG). In ovarian tissue, LH-mediated LHCGR activation is essential for ovulation and corpus luteum function, whereas hCG maintains progesterone secretion during early gestation (17–19). Interestingly, the expression and functional relevance of LHCGR in the human endometrium is controversial. Most available evidence suggests that the direct actions of LH or hCG on endometrial stromal cells are limited. Instead, hCG is believed to act predominantly through indirect pathways, such as stimulating endometrial epithelial cells to release prostaglandins (e.g., PGF2α), which subsequently promote stromal decidualization via paracrine signaling (20–22). A recent work has provided additional mechanistic insights. Women with recurrent implantation failure (RIF) have been reported to exhibit reduced endometrial LHCGR expression accompanied by impaired autophagy. *In vitro*, hCG upregulates receptivity markers and autophagy-related proteins in primary endometrial stromal cells, whereas LHCGR knockdown attenuates these effects, suggesting a functional link between LHCGR signaling, autophagy, and endometrial receptivity (23). Clinical data also suggest that moderate elevations in LH may enhance endometrial thickness and receptivity in women with intrinsically low LH, even in the absence of ovarian activity (24). In contrast, other studies have suggested that excessive or prolonged LH exposure may impair the regenerative functions of endometrial stem/progenitor cells, ultimately diminishing receptivity (25). Taken together, these findings suggest that the relationship between LH activity and endometrial receptivity is likely complex and may vary according to the magnitude, timing, and duration of LH exposure.

In our study, although the association appeared more evident in day 3 embryo transfer cycles, similar directional trends were observed in day 5 cycles, and formal interaction testing did not demonstrate a statistically significant interaction between embryo stage and LH rise (P for interaction = 0.418). Therefore, the apparent difference observed between day 3 and day 5 embryo transfer cycles should be interpreted cautiously.

Several biological explanations may account for this observation. First, cleavage-stage embryos may have a narrower implantation window and therefore could be more susceptible to subtle alterations in endometrial receptivity. In contrast, blastocysts have undergone extended *in vitro* culture and developmental selection, potentially conferring greater implantation competence. Second, blastocyst transfer occurs after a longer duration of progesterone exposure, during which endometrial receptivity may become more fully established. Given the relatively short half-life of LH compared with progesterone, any transient effect of LH elevation on the endometrium may be attenuated by the time blastocyst transfer is performed. While these biological hypotheses remain plausible, the present study does not provide definitive evidence that embryo developmental stage modifies the relationship between endogenous LH rise and reproductive outcomes. Further studies with larger sample sizes are needed to clarify whether such effect modification truly exists.

Our findings differ from several larger cohorts reporting improved outcomes among women with higher pre-P LH concentrations (9–11). In the present study, decoupling analyses suggested that basal LH may contribute importantly to the implantation association previously attributed to the LH rising ratio, whereas pre-P LH did not remain independently associated with implantation. To further explore these findings, we performed additional quartile analyses of absolute LH concentrations. Although higher basal LH quartiles showed progressively increased odds of implantation, these associations did not reach statistical significance. Likewise, pre-P LH quartiles did not demonstrate a consistent dose–response relationship across reproductive outcomes. Continuous-variable analyses of the LH rising ratio also demonstrated an association with implantation outcomes. However, these findings should be interpreted cautiously because the ratio is mathematically influenced by its denominator (basal LH concentrations), which may amplify the calculated ratio despite similar absolute pre-P LH levels. Furthermore, because basal LH constitutes the denominator of the LH rising ratio, we assessed multicollinearity using variance inflation factors, and all VIF values were below 2 (**Supplementary Table 3**), indicating no evidence of problematic multicollinearity. Nevertheless, the LH rising ratio may still partly reflect underlying basal LH levels rather than an independent effect of LH elevation.

Taken together, these findings suggest that the observed association between the LH rising ratio and reproductive outcomes is likely influenced by underlying basal LH status. Our decoupling analyses suggest that basal LH appears to contribute more strongly to implantation outcomes than pre-P LH itself, suggesting that the LH rising ratio may function primarily as a surrogate marker of low basal LH rather than reflecting an independent biological effect of LH elevation. Differences among studies may therefore reflect variations in exposure definitions, patient populations, endometrial preparation protocols, and residual baseline differences across study cohorts.

Although the physiological role of endogenous LH fluctuations during AC-FET remains incompletely understood, our findings suggest that baseline LH may be more strongly associated with reproductive outcomes than absolute pre-P LH concentrations. Nevertheless, because the LH rising ratio integrates information from both basal and pre-P LH levels, it may still provide useful information regarding the endocrine milieu before P initiation, although its observed association appears to be driven largely by basal LH. Further well-designed prospective studies incorporating serial LH measurements, embryo-quality parameters, and mechanistic investigations are required to clarify the biological significance of LH dynamics in AC-FET cycles.

### 4.1 Strength and limitations

A major strength of this study is that it explored the potential clinical significance of dynamic LH changes during artificial-cycle frozen embryo transfer (AC-FET) without pituitary suppression. Rather than focusing solely on absolute LH concentrations, we evaluated the LH rising ratio, which reflects the relative change in LH from baseline to the day before progesterone initiation. In addition, we analyzed a relatively large single-center cohort of 582 FET cycles, including both day 3 and day 5 embryo transfers. To account for repeated cycles contributed by the same woman, all reproductive outcomes were reanalyzed using generalized estimating equation (GEE) models. Furthermore, sensitivity analyses using alternative LH-rise thresholds (twofold and fourfold) yielded results consistent with the primary analysis, supporting the robustness of our findings. Also, additional analyses including alternative LH-rise thresholds, decoupling models, and quartile analyses of absolute LH concentrations were performed to evaluate the robustness and interpretation of the findings.

Several limitations should also be acknowledged. First, because of the retrospective design, residual confounding cannot be completely excluded. Variables such as smoking status, alcohol consumption, reproductive history, infertility etiology, and other potential confounders were not consistently available and therefore could not be adjusted for in the present study. Second, detailed embryo-related characteristics were not available. Although maternal age at embryo transfer and the number of embryos transferred were included in the adjusted analyses, detailed embryo-quality parameters, including the number of top-quality embryos transferred, were not available in a standardized format throughout the study period. Moreover, maternal age at oocyte retrieval, which is biologically more relevant to embryo competence in frozen embryo transfer cycles, was not consistently available in the retrospective database. Therefore, maternal age at embryo transfer was used for adjustment, which may not fully account for embryo-related aneuploidy risk. In addition, oocyte age at cryopreservation and fertilization method were not available as structured variables within the study dataset. Furthermore, the study population was relatively old, and embryos were not routinely tested for aneuploidy. Therefore, residual confounding related to embryo characteristics and embryo chromosomal status cannot be completely excluded, and the generalizability of our findings to younger populations or PGT-tested embryos should be interpreted with caution.

Third, because blood sampling was performed during routine clinical practice in the morning rather than at strictly standardized times, the influence of circadian variation on LH measurements cannot be entirely excluded. In addition, LH secretion is inherently pulsatile, and exact sampling times were not available in the retrospective database. Therefore, some degree of biological variability may have influenced individual LH measurements. Furthermore, because basal LH constitutes the denominator of the LH rising ratio, adjustment for basal LH may complicate interpretation of the independent association between the LH rising ratio and reproductive outcomes. To address this concern, multicollinearity was assessed using variance inflation factors, and all VIF values were below 2 (**Supplementary Table 5**), suggesting no evidence of problematic multicollinearity.

Fourth, we did not have serum LH measurements on the day of embryo transfer and therefore could not evaluate the persistence of LH changes throughout the implantation window. Although similar directional trends were observed in day-5 embryo transfer cycles, the relatively smaller number of cycles in the high LH-rise group may have limited statistical power. Consequently, the absence of statistically significant differences in some day 5 analyses, as well as in ongoing pregnancy and abortion outcomes, should be interpreted as inconclusive rather than evidence of a true lack of effect. Finally, live birth data were not available in the present study, and ongoing pregnancy beyond 20 weeks was therefore the highest available reproductive outcome. Because live birth is the most clinically relevant endpoint, future studies with complete live-birth follow-up are needed to validate our findings.

Fifth, additional analyses entering basal LH and pre-P LH as separate continuous variables suggested that basal LH was more strongly associated with implantation outcomes than pre-P LH. These findings suggest that the observed association between the LH rising ratio and reproductive outcomes may primarily reflect underlying basal LH status rather than an independent biological effect of LH elevation. In addition, residual baseline differences between comparison groups may have contributed to the observed associations despite multivariable adjustment. Therefore, although the LH rising ratio remained associated with implantation outcomes, its biological interpretation should be made with caution. Moreover, although the association appeared more evident in day 3 embryo transfer cycles, formal interaction testing did not demonstrate a statistically significant difference between day 3 and day 5 transfers. Consequently, the subgroup findings should be interpreted cautiously and regarded as hypothesis-generating.

## 5 Conclusions

A higher LH rising ratio before progesterone supplementation was associated with poorer reproductive outcomes in D3 AC-FET cycles. However, additional analyses suggest that this association primarily reflects underlying basal LH rather than an independent biological effect of elevated pre-P LH. No clear threshold effect was identified for either basal LH or pre-P LH. Further prospective studies are required to clarify the biological significance of LH dynamics during AC-FET cycles.

## Data Availability

The raw data supporting the conclusions of this article will be made available by the authors, without undue reservation.
