# Supplementary material for: Association of LH rising before luteal support and reproductive outcomes in artificial frozen embryo transfer cycles
Source: Front Endocrinol (Lausanne). 2026 Jul 23;17:1886113. doi: 10.3389/fendo.2026.1886113 (PMC13441795; doi:10.3389/fendo.2026.1886113)
Supplement: Supplementary file 1 [file Table1.docx]

**Supplementary Table S1. Sensitivity analyses using alternative LH-rise thresholds (2-fold, 3-fold, and 4-fold) and reproductive outcomes in AC-FET cycles**

| **LH threshold** | **Outcome** | **aOR** | **95% CI** | **P value** |
| --- | --- | --- | --- | --- |
| **≥2-fold** | **Implantation rate** | **1.59** | **1.14–2.21** | **0.006*** |
| **≥3-fold** | **Implantation rate** | **1.68** | **1.15–2.46** | **0.008*** |
| **≥4-fold** | **Implantation rate** | **2.01** | **1.17–3.43** | **0.012*** |
| **≥2-fold** | **Clinical pregnancy rate** | **0.54** | **0.33–0.88** | **0.012*** |
| **≥3-fold** | **Clinical pregnancy rate** | **0.56** | **0.34–0.95** | **0.031*** |
| **≥4-fold** | **Clinical pregnancy rate** | **0.47** | **0.24–0.89** | **0.023*** |

* Statistic method: Multivariable generalized estimating equation (GEE) analysis with an exchangeable working correlation structure.

*Alternative LH-rise thresholds were defined as follows:

2-fold analysis: Group 1, LH rising ratio <2; Group 2, LH rising ratio ≥2.

3-fold analysis: Group 1, LH rising ratio <3; Group 2, LH rising ratio ≥3.

4-fold analysis: Group 1, LH rising ratio <4; Group 2, LH rising ratio ≥4.

^*^ p < 0.05 considered statistically significant.

Supplementary Table S1 presents sensitivity analyses evaluating the association between endogenous LH rise and reproductive outcomes using alternative LH-rise thresholds of 2-fold, 3-fold, and 4-fold. Multivariable generalized estimating equation (GEE) models were applied to account for repeated cycles contributed by the same woman. The associations between elevated LH rise and both implantation rate and clinical pregnancy rate remained directionally consistent across all tested thresholds, supporting the robustness of the primary findings and indicating that the observed associations were not dependent on the specific cutoff selected.
