# Supplementary material for: Association of LH rising before luteal support and reproductive outcomes in artificial frozen embryo transfer cycles
Source: Front Endocrinol (Lausanne). 2026 Jul 23;17:1886113. doi: 10.3389/fendo.2026.1886113 (PMC13441795; doi:10.3389/fendo.2026.1886113)
Supplement: Supplementary file 2 [file Table2.docx]

**Supplementary Table S2. Independent associations of basal LH and pre-progesterone LH with reproductive outcomes in AC-FET cycles**

| **Variable** | **Outcome** | **aOR** | **95% CI** | **P value** |
| --- | --- | --- | --- | --- |
| **Basal LH** | **Implantation rate** | **1.09** | **1.02–1.18** | **0.017*** |
| **Pre-P LH** | **Implantation rate** | **0.99** | **0.96–1.02** | **0.482** |
| **Basal LH** | **Clinical pregnancy rate** | **0.92** | **0.84–1.02** | **0.108** |
| **Pre-P LH** | **Clinical pregnancy rate** | **1.01** | **0.98–1.05** | **0.512** |

* Statistic method: Multivariable generalized estimating equation (GEE) analysis with an exchangeable working correlation structure.

*Basal LH and pre-progesterone LH were entered simultaneously as continuous variables in the same model to evaluate their independent associations with reproductive outcomes.

*Implantation rate was analyzed using a binomial GEE model with the number of gestational sacs specified as events and the number of embryos transferred specified as trials.

*The denominators for clinical pregnancy rate was the number of embryo transfer cycles.
^*^ p < 0.05 considered statistically significant.

Supplementary Table S2 presents additional analyses in which basal LH and pre-progesterone LH were entered simultaneously as continuous variables in multivariable GEE models. These analyses were performed to disentangle the respective contributions of baseline LH status and pre-progesterone LH levels to reproductive outcomes. Basal LH, but not pre-progesterone LH, was independently associated with implantation rate, suggesting that baseline LH status may partially contribute to the association observed between the LH rising ratio and reproductive outcomes.
