# Supplementary material for: Association of LH rising before luteal support and reproductive outcomes in artificial frozen embryo transfer cycles
Source: Front Endocrinol (Lausanne). 2026 Jul 23;17:1886113. doi: 10.3389/fendo.2026.1886113 (PMC13441795; doi:10.3389/fendo.2026.1886113)
Supplement: Supplementary file 3 [file Table3.docx]

**Supplementary Table S3. Association between absolute LH quartiles and reproductive outcomes in AC-FET cycles**

A. **Pre-progesterone LH Quartiles**

| **Quartile** | **LH range**  **(mIU/mL)** | **Implantation rate OR (95%CI)** | **P value** | **Clinical pregnancy rate OR (95%CI)** | **P value** | **Abortion rate OR (95%CI)** | **P value** | **Ongoing pregnancy rate OR (95%CI)** | **P value** |
| --- | --- | --- | --- | --- | --- | --- | --- | --- | --- |
| **Q1** | **<5.47** | **Ref** | **-** | **Ref** | **-** | **Ref** | **-** | **Ref** | **-** |
| **Q2** | **5.47-10.09** | **0.68 (0.43-1.07)** | **0.098** | **2.41 (1.25-4.65)** | **0.008*** | **2.22 (0.35-14.18)** | **0.398** | **2.01 (0.97-4.17)** | **0.060** |
| **Q3** | **10.09-15.19** | **0.70 (0.45-1.10)** | **0.124** | **2.28 (1.18-4.39)** | **0.014*** | **0.65 (0.06-7.49)** | **0.726** | **1.53 (0.76-3.10)** | **0.229** |
| **Q4** | **≥15.19** | **0.69 (0.44-1.09)** | **0.114** | **1.99 (1.05-3.78)** | **0.035*** | **0.69 (0.06-7.59)** | **0.761** | **1.26 (0.63-2.49)** | **0.513** |

B. **Basal LH Quartiles**

| **Quartile** | **LH range (mIU/mL)** | **Implantation rate OR (95%CI)** | **P value** |
| --- | --- | --- | --- |
| **Q1** | **<3.32** | **Ref** | **-** |
| **Q2** | **3.32-4.92** | **1.20 (0.74-1.96)** | **0.463** |
| **Q3** | **4.91-6.42** | **1.46 (0.92-2.32)** | **0.105** |
| **Q4** | **≥6.42** | **1.51 (0.96-2.38)** | **0.076** |

* Quartiles were defined according to the distributions of basal LH and pre-progesterone LH concentrations within the study cohort.

*Odds ratios (ORs) and 95% confidence intervals (CIs) were estimated using generalized estimating equation (GEE) models to account for repeated treatment cycles within the same woman.

*The Quartiles were defined according to the distribution of LH concentrations within the study cohort. Q1 represents values at or below the 25th percentile, Q2 values above the 25th percentile and up to the 50th percentile, Q3 values above the 50th percentile and up to the 75th percentile, and Q4 values above the 75th percentile. The lowest quartile (Q1) served as the reference category in all analyses.

*Implantation was modeled as the number of intrauterine gestational sacs relative to the number of embryos transferred.
* p < 0.05 considered statistically significant.
