# Supplementary material for: Association of LH rising before luteal support and reproductive outcomes in artificial frozen embryo transfer cycles
Source: Front Endocrinol (Lausanne). 2026 Jul 23;17:1886113. doi: 10.3389/fendo.2026.1886113 (PMC13441795; doi:10.3389/fendo.2026.1886113)
Supplement: Supplementary file 4 [file Table4.docx]

**Supplementary Table S4. Continuous analysis of LH rising ratio and reproductive outcomes using GEE models**

| **Outcome** | **OR per 1-unit increase in LH rising ratio** | **95% CI** | **P value** |
| --- | --- | --- | --- |
| **Implantation rate** | **0.876** | **0.802–0.958** | **0.004*** |
| **Clinical pregnancy rate** | **1.176** | **1.024–1.350** | **0.022*** |
| **Abortion rate** | **0.877** | **0.605–1.271** | **0.488** |
| **Ongoing pregnancy rate** | **1.087** | **0.972–1.215** | **0.145** |

* Statistic method: Multivariable generalized estimating equation (GEE) analysis with an exchangeable working correlation structure.

*ORs were estimated using generalized estimating equation (GEE) models accounting for repeated treatment cycles. LH rising ratio was analyzed as a continuous variable.

*Implantation rate was analyzed using a binomial GEE model with the number of gestational sacs specified as events and the number of embryos transferred specified as trials.

*The denominators for clinical pregnancy rate was the number of embryo transfer cycles.
^*^ p < 0.05 considered statistically significant.
