# Supplementary material for: Association of LH rising before luteal support and reproductive outcomes in artificial frozen embryo transfer cycles
Source: Front Endocrinol (Lausanne). 2026 Jul 23;17:1886113. doi: 10.3389/fendo.2026.1886113 (PMC13441795; doi:10.3389/fendo.2026.1886113)
Supplement: Supplementary file 5 [file Table5.docx]

**Supplementary Table S5. Assessment of multicollinearity among covariates included in the multivariable GEE models**

| **Variable** | **D3 VIF** | **D5 VIF** |
| --- | --- | --- |
| **LH rising ratio** | **1.297** | **1.201** |
| **BMI** | **1.162** | **1.183** |
| **AMH** | **-** | **1.174** |
| **Basal E2** | **-** | **1.173** |
| **Basal LH** | **1.493** | **1.345** |
| **Basal P** | **1.127** | **1.330** |
| **Basal FSH** | **1.242** | **-** |
| **Pre-P E2** | **1.099** | **1.098** |
| **Pre-P P** | **1.151** | **1.233** |
| **Endometrial thickness** | **1.039** | **1.044** |
| **Number of embryos transferred** | **1.080** | **1.101** |

* Statistic method: VIFs were calculated to assess multicollinearity among predictors included in the multivariable GEE models. All VIFs were <2, indicating no evidence of problematic multicollinearity.

* Age was specified as the dependent variable solely for the purpose of VIF calculation in SPSS; therefore, a VIF was not generated.

* VIF: variance inflation factor, BMI: body mass index, AMH: anti-Mullerian hormone, E2: estradiol, LH: luteinizing hormone, P4: progesterone, FSH: follicle-stimulating hormone, pre-P: the day before progesterone supplementation
